# 91 Psychometric Properties of the Brisbane Burn Scar Impact Profile for Adult Patients

**DOI:** 10.1093/jbcr/irae036.090

**Published:** 2024-04-17

**Authors:** Martin R Buta, Sarah Findeisen, Pengsheng Ni, Lewis E Kazis, Sean Hickey, Jonathan S Friedstat, John T Schulz, Colleen M Ryan, Jeffrey C Schneider, Jeremy Goverman

**Affiliations:** Massachusetts General Hospital, Boston, MA; Boston University School of Public Health, Boston, MA; Department of Health Law, Policy and Management, Massachusetts General Hospital/Shriners Children's, Boston, MA; Spaulding Rehabilitation Hospital/Harvard Medical School, Boston, MA; Massachusetts General Hospital, Boston, MA; Boston University School of Public Health, Boston, MA; Department of Health Law, Policy and Management, Massachusetts General Hospital/Shriners Children's, Boston, MA; Spaulding Rehabilitation Hospital/Harvard Medical School, Boston, MA; Massachusetts General Hospital, Boston, MA; Boston University School of Public Health, Boston, MA; Department of Health Law, Policy and Management, Massachusetts General Hospital/Shriners Children's, Boston, MA; Spaulding Rehabilitation Hospital/Harvard Medical School, Boston, MA; Massachusetts General Hospital, Boston, MA; Boston University School of Public Health, Boston, MA; Department of Health Law, Policy and Management, Massachusetts General Hospital/Shriners Children's, Boston, MA; Spaulding Rehabilitation Hospital/Harvard Medical School, Boston, MA; Massachusetts General Hospital, Boston, MA; Boston University School of Public Health, Boston, MA; Department of Health Law, Policy and Management, Massachusetts General Hospital/Shriners Children's, Boston, MA; Spaulding Rehabilitation Hospital/Harvard Medical School, Boston, MA; Massachusetts General Hospital, Boston, MA; Boston University School of Public Health, Boston, MA; Department of Health Law, Policy and Management, Massachusetts General Hospital/Shriners Children's, Boston, MA; Spaulding Rehabilitation Hospital/Harvard Medical School, Boston, MA; Massachusetts General Hospital, Boston, MA; Boston University School of Public Health, Boston, MA; Department of Health Law, Policy and Management, Massachusetts General Hospital/Shriners Children's, Boston, MA; Spaulding Rehabilitation Hospital/Harvard Medical School, Boston, MA; Massachusetts General Hospital, Boston, MA; Boston University School of Public Health, Boston, MA; Department of Health Law, Policy and Management, Massachusetts General Hospital/Shriners Children's, Boston, MA; Spaulding Rehabilitation Hospital/Harvard Medical School, Boston, MA; Massachusetts General Hospital, Boston, MA; Boston University School of Public Health, Boston, MA; Department of Health Law, Policy and Management, Massachusetts General Hospital/Shriners Children's, Boston, MA; Spaulding Rehabilitation Hospital/Harvard Medical School, Boston, MA; Massachusetts General Hospital, Boston, MA; Boston University School of Public Health, Boston, MA; Department of Health Law, Policy and Management, Massachusetts General Hospital/Shriners Children's, Boston, MA; Spaulding Rehabilitation Hospital/Harvard Medical School, Boston, MA

## Abstract

**Introduction:**

Many burn injuries result in a prolonged recovery involving extensive therapy and surgical reconstruction. Burn patients experience a broad range of symptoms and acute symptoms can develop into chronic conditions that can significantly impact function and overall quality of life. Hypertrophic scars, a complication from burn injury, can lead to contractures, tightness, pain, pruritis, altered appearance, and impaired psychosocial well-being. The Brisbane Burn Scar Impact Profile (BBSIP) is a patient-reported health-related quality of life survey designed to assess the impact of burn scars in children and adults. In this study, we aimed to assess the reliability and factor structure of the BBSIP in adult patients with hypertrophic burn scars undergoing reconstruction and/or laser treatment.

**Methods:**

A prospective cohort study enrolled 113 adult patients with hypertrophic burn scars undergoing reconstruction, including skin grafts, and/or laser treatment at a North American major tertiary care center. Patient demographics, percent total body surface area (TBSA) burned, and burn etiologies were obtained. A total of 238 surveys were collected after a reconstructive or laser treatment. An exploratory and confirmatory factor analysis was carried out on the seven sub-domains of the BBSIP to determine the reliability of each. For each sub-domain, we determined the acceptable model fit with a comparative fit index (CFI) >0.9 and a root mean square error of approximation (RMSEA) < 0.1. We considered a sub-domain to have acceptable reliability if the omega reliability or coefficient omega hierarchical was greater than 0.8.

**Results:**

Of the 113 patients (59 female, 54 male), 61 (54%) completed the survey only once, 20 (17.7%) completed 2 surveys, and 16 (14.2%) completed 3 surveys. For the burn patients, most sub-domains in the BBSIP (sensory symptoms, work/daily activities, relationships/social interactions, appearance, emotional reactions, and physical symptoms) presented with a unidimensional scale (CFI>0.9 and RMSEA < 0.1) and had an acceptable reliability (omega reliability or coefficient omega hierarchical ranged from 0.8 to 0.94) after eliminating select items from the profile that were redundant. The “overall impact” sub-domain presented as a multidimensional scale and was also found to have acceptable reliability.

**Conclusions:**

This study demonstrates acceptable reliability of the BBSIP in adult patients with hypertrophic burn scars undergoing reconstruction and/or laser treatment. To strengthen BBSIP reliability, within each sub-domain (except “overall impact”), we recommend deleting misfit items and pairing others. This modification would decrease the redundancy of questions and ensure the survey more reliably assesses the impact of burn scars.

**Applicability of Research to Practice:**

This study provides recommended changes to the BBSIQ that will improve its reliability in the clinical setting.

